# Association Between Documented Hypertension and In-Hospital Mortality Among Critically Ill Adults: A Retrospective Cohort Study Using the Medical Information Mart for Intensive Care IV Database

**DOI:** 10.7759/cureus.113156

**Published:** 2026-07-22

**Authors:** Angel Dockery, Goitom Abraha, Mohammed Raaid O Musa, Nonso Ariahu, Franklin I Nnorom

**Affiliations:** 1 Anesthesiology, Savanna-La-Mar Public General Hospital, Westmoreland, JAM; 2 Internal Medicine, University of New Mexico Hospital, Albuquerque, USA; 3 Internal Medicine, Harlem Hospital Center, New York, USA; 4 Medicine and Surgery, College of Medicine, University of Lagos, Lagos, NGA; 5 Sterile Processing, Wellstar Health System, Augusta, USA

**Keywords:** critical illness, hypertension, in-hospital mortality, intensive care unit, mimic-iv, sofa score

## Abstract

Background

Hypertension is one of the most common chronic conditions worldwide and is frequently encountered among patients admitted to ICUs. Although hypertension is a well-established risk factor for cardiovascular morbidity and mortality, its association with outcomes among critically ill patients remains incompletely understood.

Objective

To determine whether documented hypertension is associated with in-hospital mortality among critically ill adults admitted to ICUs.

Methods

This retrospective cohort study used data from the Medical Information Mart for Intensive Care IV (MIMIC-IV) version 3.1 database. Adult patients aged 18 years and older with a first ICU admission were included. Hypertension was identified using International Classification of Diseases, Ninth Revision (ICD-9), and Tenth Revision (ICD-10) diagnostic codes. The primary outcome was in-hospital mortality. Multivariable logistic regression was used to evaluate the association between hypertension and mortality after adjustment for age, sex, race or ethnicity, Charlson Comorbidity Index (CCI), Sequential Organ Failure Assessment (SOFA) score, and mechanical ventilation. A sensitivity analysis was performed using the Oxford Acute Severity of Illness Score (OASIS).

Results

A total of 65,339 critically ill adults were included in the final analysis, of whom 41,193 (63.1%) had documented hypertension. In-hospital mortality occurred in 7,078 patients (10.8%). Patients who died were older and had higher CCI, SOFA, and OASIS values than survivors (all p < 0.001). After adjustment for potential confounders, hypertension was associated with lower odds of in-hospital mortality (adjusted OR, 0.67; 95% confidence interval, 0.63 to 0.71; p < 0.001). Higher age, CCI, SOFA score, and mechanical ventilation were associated with increased odds of mortality. Similar findings were observed in the sensitivity analysis using the OASIS (adjusted OR, 0.70; 95% CI, 0.66 to 0.74; p < 0.001).

Conclusion

Documented hypertension was associated with lower odds of in-hospital mortality among critically ill adults after adjustment for demographic and clinical characteristics. Comorbidity burden, illness severity, and mechanical ventilation remained important factors associated with mortality. Further studies are needed to examine the influence of hypertension severity, blood pressure control, and treatment patterns on outcomes among critically ill patients.

## Introduction

Hypertension is one of the most common chronic diseases worldwide and remains a major contributor to cardiovascular morbidity, mortality, and healthcare expenditure. It is estimated to affect more than 1.2 billion adults globally, and elevated blood pressure is responsible for approximately 10.8 million deaths each year, making it one of the leading modifiable risk factors for premature mortality worldwide [1,2]. Despite advances in prevention and treatment, the global burden of hypertension continues to increase because of population aging, unhealthy lifestyles, and the rising prevalence of obesity and metabolic disorders [3]. Consequently, hypertension represents a major public health challenge and is frequently encountered among patients requiring hospitalization and intensive care [4].

Patients in the ICU are at increased risk of death due to physiological instability, multi-organ failure, and complex comorbidities [5]. Identifying the factors that predict mortality among ICU patients is critical for improving risk stratification, assisting clinical decision-making, and improving patient outcomes [6]. It is also important to investigate the influence of pre-existing chronic conditions on ICU survival [7]. Understanding how chronic diseases affect critical care outcomes may help clinicians recognize patients at increased risk and develop focused management strategies [8].

Hypertension is common among ICU patients, who often have numerous chronic conditions, many of which may affect their outcomes [9]. Long-standing hypertension produces both structural and functional changes in many organs, including left ventricular hypertrophy, vascular remodeling, endothelial dysfunction, and reduced renal function [10, 11]. These pathophysiological changes may decrease the physiological reserve of these organs and predispose patients to a greater likelihood of poor outcomes during critical illness [12]. Comorbidities such as diabetes mellitus may further complicate their care and increase their chances of dying [13, 14].

The relationship between hypertension and mortality has produced mixed results across numerous studies. Some studies have reported that hypertension is associated with increased mortality because of its contribution to cardiovascular complications and end-organ damage [15,16]. In contrast, studies among critically ill patients have reported similar or even lower mortality among patients with documented hypertension than among those without hypertension after adjustment for demographic and clinical characteristics [17,18]. These inconsistent findings may reflect differences in study populations, illness severity, antihypertensive treatment, and methodological approaches [19]. Consequently, it remains unclear whether documented hypertension is independently associated with in-hospital mortality among critically ill adults.

The Medical Information Mart for Intensive Care IV (MIMIC-IV) is a large, publicly available electronic health record database containing detailed demographic, clinical, diagnostic, treatment, and outcome information for patients admitted to ICUs at Beth Israel Deaconess Medical Center in the United States [20]. Its large sample size and comprehensive clinical data allow adjustment for patient characteristics, comorbidities, and illness severity, making it well suited for investigating factors associated with outcomes among critically ill adults.

Although hypertension is one of the most common chronic conditions among critically ill patients, its independent association with in-hospital mortality remains uncertain. Clarifying this relationship may improve risk stratification and provide greater insight into the role of hypertension in critical illness. Therefore, this study aimed to examine the association between documented hypertension and in-hospital mortality among critically ill adults using the MIMIC-IV database.

## Materials and methods

Study design and data source

This retrospective cohort study was conducted using data from the MIMIC-IV database, version 3.1, which includes ICU admissions at Beth Israel Deaconess Medical Center between 2008 and 2022. MIMIC-IV is a large, publicly available clinical database containing de-identified health information for patients admitted to Beth Israel Deaconess Medical Center in Boston, Massachusetts, United States [21]. The database includes detailed information on demographics, diagnoses, procedures, laboratory measurements, vital signs, medications, ICU admissions, and hospital outcomes. Data extraction was performed using Google BigQuery after completion of the required Collaborative Institutional Training Initiative certification and PhysioNet data use agreement.

Study population

**Figure 1:**
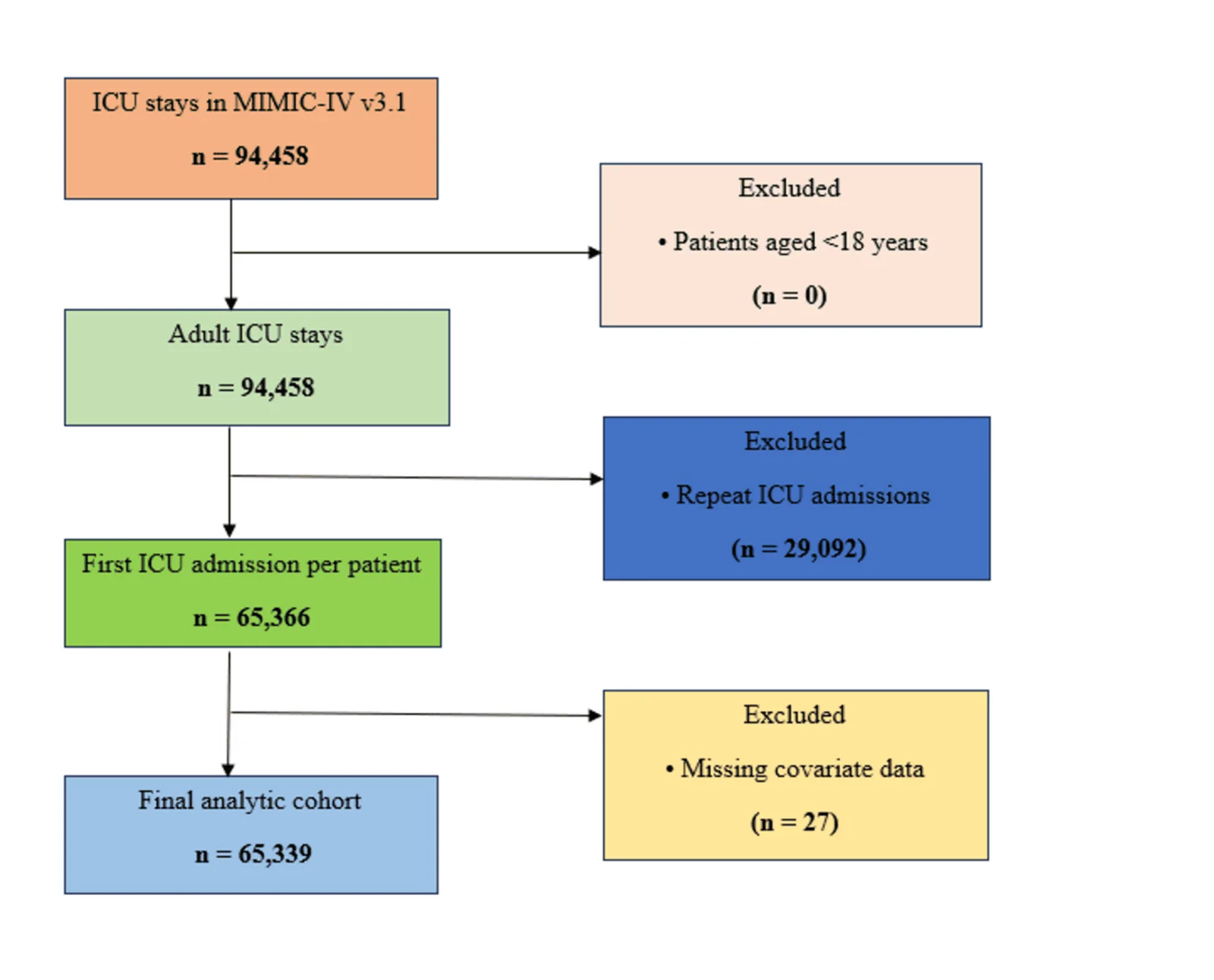
Flow diagram of study population selection from the MIMIC-IV database. MIMIC-IV: Medical Information Mart for Intensive Care IV.

The study population consisted of adult patients admitted to an ICU during the study period represented in the MIMIC-IV database. One ICU admission per patient was retained by selecting the first ICU admission to ensure independence of observations. A total of 65,366 patients comprised the initial study cohort. After excluding 27 patients with missing data in variables included in the multivariable analyses, the final analytic cohort consisted of 65,339 patients (Figure 1).

Variables and measures

The primary exposure was documented hypertension. Hypertension was identified using International Classification of Diseases, Ninth Revision (ICD-9), and Tenth Revision (ICD-10) diagnosis codes recorded during the index hospitalization. Specifically, the exposure included ICD-9 codes 401-405, representing essential hypertension (401), hypertensive heart disease (402), hypertensive chronic kidney disease (403), hypertensive heart and chronic kidney disease (404), and secondary hypertension (405), as well as ICD-10 codes I10-I13 and I15-I16, representing essential hypertension (I10), hypertensive heart disease (I11), hypertensive chronic kidney disease (I12), hypertensive heart and chronic kidney disease (I13), secondary hypertension (I15), and hypertensive crisis (I16). These diagnoses were combined to capture documented hypertension and related hypertensive disorders recorded during the index hospitalization. Patients meeting any of these criteria were classified as having hypertension, while all remaining patients were classified as not having hypertension.

The primary outcome was in-hospital mortality, defined using the hospital_expire_flag variable from the admissions table. Patients who died during the index hospitalization were coded as having experienced the outcome.

Covariates were selected based on their clinical relevance and established association with mortality among critically ill patients. Demographic variables included age, sex, and race or ethnicity. Race and ethnicity categories were collapsed into White, Black, Hispanic, Asian, Other, and Unknown groups. Comorbidity burden was assessed using the Charlson Comorbidity Index (CCI) derived from diagnostic information. Severity of illness was assessed using the Sequential Organ Failure Assessment (SOFA) score and the Oxford Acute Severity of Illness Score (OASIS). For SOFA, the maximum SOFA score recorded during the first 24 hours of ICU admission was used. Mechanical ventilation was defined as the presence of invasive mechanical ventilation at any time during the ICU stay. Because the timing of mechanical ventilation relative to ICU admission was not restricted to the first 24 hours, this variable may not always represent baseline illness severity. ICU length of stay was measured in days using information from the icustays table.

Missing data

Missing data were minimal across study variables. The SOFA score had 0.02% missing values, and ICU length of stay had 0.02% missing values. No missing values were identified for age, sex, race or ethnicity, hypertension status, mortality, OASIS score, CCI, or mechanical ventilation status. Given the very low level of missingness, a complete case analysis was performed, and observations with missing values in variables included in the regression models were excluded.

Statistical analysis

All statistical analyses were performed using Stata version 18 [22]. Given the large sample size, continuous variables were summarized as mean ± SD, as these measures provide stable summary estimates under the Central Limit Theorem. Categorical variables were summarized using frequencies and column percentages. Baseline characteristics were compared according to in-hospital mortality status. Group comparisons for continuous variables were conducted using independent-sample t tests, while categorical variables were compared using chi-square tests.

Before fitting the multivariable logistic regression model, the assumptions of logistic regression were evaluated. The linearity of continuous variables in the logit was assessed for age, CCI, SOFA, and OASIS, and the assumption was satisfied. Multicollinearity and influential observations were also assessed, and no significant violations were identified.

Multivariable logistic regression models were subsequently fitted to estimate adjusted odds ratios and 95% CIs while controlling for age, sex, race or ethnicity, CCI, SOFA score, and invasive mechanical ventilation. A sensitivity analysis was performed using OASIS instead of SOFA, without separately adjusting for age and mechanical ventilation, both of which are components of OASIS, in the adjusted model to examine the robustness of the findings using an alternative measure of illness severity. Multicollinearity among independent variables was assessed using variance inflation factors. Variance inflation factor values ranged from 1.02 to 5.01, with a mean variance inflation factor of 2.69, indicating no evidence of problematic multicollinearity. Statistical significance was defined as a two-sided p value less than 0.05.

Ethical considerations

MIMIC-IV contains de-identified patient information and complies with the Health Insurance Portability and Accountability Act Safe Harbor requirements. Access to the database is granted only to users who complete the required human research protection training and agree to the PhysioNet data use agreement. Because all data were fully de-identified, the study did not involve direct contact with patients, and no additional informed consent was required. This study was conducted in accordance with the ethical and data use requirements governing access to the MIMIC-IV database.

## Results

Table 1 presents the baseline characteristics of critically ill adults according to documented hypertension status. The findings compare the demographic and clinical characteristics of critically ill adults according to documented hypertension status. Patients with documented hypertension were older and had a greater burden of comorbidities than those without hypertension. They also had higher illness severity, reflected by higher SOFA and OASIS scores, and a slightly longer ICU length of stay. Patients with documented hypertension were more likely to receive mechanical ventilation and had a slightly higher proportion of in-hospital mortality. Differences were also observed in sex and race or ethnicity between the two groups. Detailed descriptive statistics and results of the group comparisons are presented in Table 1.

**Table 1:** Baseline characteristics of critically ill adults according to documented hypertension status (N = 65,339). Continuous variables are presented as mean (SD), and categorical variables are presented as frequency (column percentage). Differences between patients with and without documented hypertension were assessed using independent-sample t tests for continuous variables and chi-square tests for categorical variables. An asterisk (*) indicates statistical significance at p < 0.05. A dash (-) indicates that the cell was intentionally left blank. The table was generated by the co-authors using Stata version 18 [22]. SOFA: Sequential Organ Failure Assessment; OASIS: Oxford Acute Severity of Illness Score.

Variable	No documented hypertension (n = 24,146)	Documented hypertension (n = 41,193)	Test statistic	p value
Age, years, mean (SD)	54.64 (18.50)	68.56 (13.78)	t = -109.46	<0.001*
Charlson Comorbidity Index, mean (SD)	3.36 (2.87)	5.41 (2.82)	t = -89.37	<0.001*
SOFA score, mean (SD)	4.36 (3.83)	4.87 (3.52)	t = -17.39	<0.001*
OASIS score, mean (SD)	29.37 (8.74)	31.23 (8.37)	t = -26.90	<0.001*
ICU length of stay, days, mean (SD)	3.46 (5.47)	3.56 (5.07)	t = -2.46	0.014*
In-hospital mortality, n (%)	-	-	χ²(1) = 12.15	<0.001*
Survived	21,664 (89.72%)	36,597 (88.84%)	-	-
Died	2,482 (10.28%)	4,596 (11.16%)	-	-
Sex, n (%)	-	-	χ²(1) = 40.21	<0.001*
Female	10,971 (45.44%)	17,666 (42.89%)	-	-
Male	13,175 (54.56%)	23,527 (57.11%)	-	-
Race or ethnicity, n (%)	-	-	χ²(5) = 321.37	<0.001*
Asian	830 (3.44%)	1,150 (2.79%)	-	-
Black	1,665 (6.90%)	4,350 (10.56%)	-	-
Hispanic	974 (4.03%)	1,375 (3.34%)	-	-
Other	989 (4.10%)	1,554 (3.77%)	-	-
Unknown	3,892 (16.12%)	5,703 (13.84%)	-	-
White	15,796 (65.42%)	27,061 (65.69%)	-	-
Mechanical ventilation, n (%)	-	-	χ²(1) = 43.57	<0.001*
No	15,302 (63.37%)	25,034 (60.77%)	-	-
Yes	8,844 (36.63%)	16,159 (39.23%)	-	-

Table 2 presents the results of the multivariable logistic regression analysis examining the association between documented hypertension and in-hospital mortality after adjustment for demographic characteristics, comorbidity burden, illness severity, and mechanical ventilation.

**Table 2:** Multivariable logistic regression analysis of factors associated with in-hospital mortality among critically ill adults (n = 65,339). Multivariable logistic regression was used to estimate adjusted odds ratios and 95% CIs for factors associated with in-hospital mortality. The model was adjusted for hypertension status, age, sex, race or ethnicity, Charlson Comorbidity Index, Sequential Organ Failure Assessment score, and mechanical ventilation. Reference categories were no documented hypertension, male sex, Asian race or ethnicity, and no mechanical ventilation. Odds ratios greater than 1 indicate higher odds of in-hospital mortality, whereas odds ratios less than 1 indicate lower odds of in-hospital mortality. An asterisk (*) indicates statistical significance at p < 0.05. A dash (-) indicates that the cell was intentionally left blank. The table was generated by the co-authors using Stata version 18 [22]. SOFA: Sequential Organ Failure Assessment.

Variable	Adjusted odds ratio (95% CI)	p value
Documented hypertension	-	-
Documented hypertension vs no documented hypertension	0.67 (0.63-0.71)	<0.001*
Age, per year increase	1.02 (1.02-1.02)	<0.001*
Sex	-	-
Female vs male	1.31 (1.23-1.38)	<0.001*
Race or ethnicity	-	-
Black vs Asian	0.87 (0.72-1.05)	0.136
Hispanic vs Asian	0.76 (0.60-0.97)	0.026*
Other vs Asian	0.92 (0.74-1.15)	0.456
Unknown vs Asian	1.83 (1.54-2.17)	<0.001*
White vs Asian	0.85 (0.72-1.00)	0.048*
Charlson Comorbidity Index, per point increase	1.16 (1.15-1.17)	<0.001*
Sequential Organ Failure Assessment score, per point increase	1.30 (1.29-1.31)	<0.001*
Mechanical ventilation	-	-
Yes vs no	1.22 (1.14-1.30)	<0.001*

After adjustment for demographic characteristics, comorbidities, illness severity, and mechanical ventilation, patients with documented hypertension had lower adjusted odds of in-hospital mortality than those without documented hypertension (aOR = 0.67, 95% CI: 0.63-0.71). Increasing age, greater comorbidity burden, higher SOFA score, female sex, and receipt of mechanical ventilation were all independently associated with higher odds of in-hospital mortality. Mortality also differed by race and ethnicity, with Hispanic patients demonstrating lower odds of mortality and patients with unknown race or ethnicity demonstrating higher odds compared with Asian patients. Detailed adjusted effect estimates are presented in Table 2.

Table 3 presents the results of the sensitivity analysis in which the SOFA score was replaced with the OASIS score to evaluate whether the observed association between documented hypertension and in-hospital mortality remained consistent when an alternative measure of illness severity was used.

**Table 3:** Sensitivity analysis using the OASIS to evaluate the robustness of the association between documented hypertension and in-hospital mortality. Multivariable logistic regression was performed using the OASIS in place of the SOFA score. The model was adjusted for hypertension status, sex, race or ethnicity, Charlson Comorbidity Index, and Oxford Acute Severity of Illness Score. Reference categories were no documented hypertension, male sex, and Asian race or ethnicity. Odds ratios greater than 1 indicate higher odds of in-hospital mortality, whereas odds ratios less than 1 indicate lower odds of in-hospital mortality. An asterisk (*) indicates statistical significance at p < 0.05. A dash (-) indicates that the cell was intentionally left blank. The table was generated by the co-authors using Stata version 18 [22]. aOR: adjusted odds ratio; OASIS: Oxford Acute Severity of Illness Score; SOFA: Sequential Organ Failure Assessment.

Variable	Adjusted odds ratio (95% CI)	p value
Documented hypertension	-	-
Documented hypertension vs no documented hypertension	0.70 (0.66-0.74)	<0.001*
Sex	-	-
Female vs male	1.04 (0.98-1.09)	0.21
Race or ethnicity	-	-
Black vs Asian	0.81 (0.68-0.97)	0.024*
Hispanic vs Asian	0.82 (0.65-1.03)	0.093
Other vs Asian	0.98 (0.79-1.21)	0.848
Unknown vs Asian	1.83 (1.55-2.17)	<0.001*
White vs Asian	0.84 (0.71-0.99)	0.033*
Charlson Comorbidity Index, per point increase	1.19 (1.18-1.20)	<0.001*
Oxford Acute Severity of Illness Score, per point increase	1.12 (1.11-1.12)	<0.001*

In the sensitivity analysis using OASIS as the measure of illness severity, documented hypertension remained independently associated with lower in-hospital mortality (aOR = 0.70, 95% CI: 0.66-0.74; p < 0.001), consistent with the primary analysis. Higher CCI and OASIS scores were independently associated with increased odds of in-hospital mortality.

**Figure 2:**
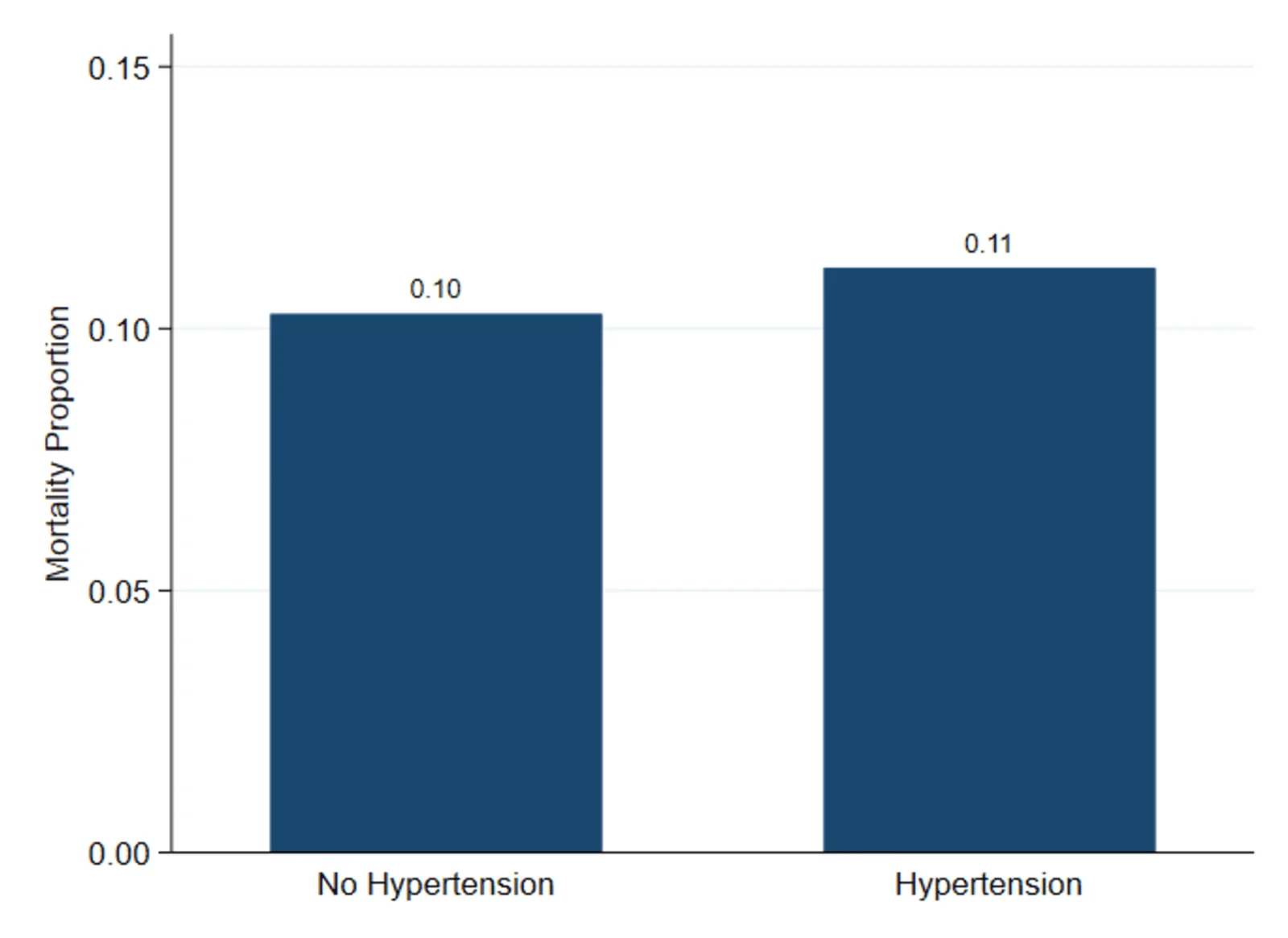
In-hospital mortality proportion by hypertension status.

Figure 2 illustrates the in-hospital mortality proportion among critically ill adults according to hypertension status.

The figure shows that the proportion of in-hospital mortality was higher among patients with documented hypertension than among those without hypertension. Patients with hypertension had a mortality proportion of approximately 0.11, while patients without hypertension had a mortality proportion of approximately 0.10. Although the absolute difference between the two groups was modest, patients with hypertension experienced a higher unadjusted mortality proportion. This pattern is consistent with the descriptive findings presented in Table 1, where patients with hypertension had a slightly higher in-hospital mortality proportion than those without hypertension.

## Discussion

This study examined the association between documented hypertension and in-hospital mortality among critically ill adults admitted to intensive care units using data from the MIMIC-IV database. The findings showed that documented hypertension was common in this population, affecting more than half of the study cohort. Although patients with documented hypertension had slightly higher crude in-hospital mortality than those without documented hypertension, they were also older and had a greater burden of comorbidities and higher illness severity at baseline. After adjustment for age, sex, race or ethnicity, comorbidity burden, illness severity, and mechanical ventilation, documented hypertension was statistically associated with lower odds of in-hospital mortality. The analysis also identified greater comorbidity burden, higher illness severity, and mechanical ventilation as factors associated with increased mortality. These findings contribute to the ongoing discussion regarding the relationship between documented hypertension and outcomes among critically ill populations.

Hypertension remains one of the most prevalent chronic conditions worldwide and is a major contributor to cardiovascular and renal disease [1-4,15]. Previous studies have shown that patients with hypertension frequently present with multiple chronic conditions and increased healthcare utilization [2,4,11,12]. Recent work among critically ill patients with hypertension has also demonstrated that severity of illness and coexisting medical conditions play an important role in mortality prediction [8]. The lower adjusted odds of mortality observed among patients with documented hypertension may reflect differences in baseline clinical characteristics, long-term treatment exposure, patterns of ICU admission, or residual differences in patient case mix that were not fully captured by routinely collected clinical variables. The higher crude mortality observed among patients with documented hypertension was largely explained after adjustment for differences in age, comorbidity burden, and illness severity, suggesting that these baseline characteristics accounted for much of the apparent unadjusted difference. The findings indicate that the relationship between documented hypertension and mortality among critically ill adults is complex and should be interpreted cautiously as an adjusted statistical association rather than evidence of a protective causal effect.

Current United States hypertension guidelines emphasize early identification, risk assessment, and effective blood pressure control to reduce cardiovascular complications and long-term adverse outcomes [4,10]. The 2017 American College of Cardiology and American Heart Association guideline recommends comprehensive cardiovascular risk evaluation and individualized management strategies for adults with hypertension [10]. Similarly, the JACC Health Promotion Series highlights the importance of prevention, treatment adherence, and risk factor modification throughout the continuum of care [4]. Although these guidelines primarily focus on outpatient prevention and management, the present findings reinforce the importance of considering chronic cardiovascular conditions when evaluating critically ill patients. The high prevalence of documented hypertension observed in this cohort reflects the substantial burden of this condition within populations requiring intensive care services.

Several biological and clinical factors may help explain the observed associations. Chronic hypertension is associated with structural and functional changes in the cardiovascular system, including arterial remodeling, endothelial dysfunction, and altered vascular responsiveness [12,13]. Hypertension is also closely linked to heart failure, chronic kidney disease, and other conditions that may influence outcomes during critical illness [2,11]. In this study, patients with documented hypertension had a substantially greater comorbidity burden than those without documented hypertension, suggesting that underlying chronic disease burden may be an important contributor to mortality risk. The association between higher comorbidity burden and mortality observed in the adjusted models is consistent with the expectation that patients with multiple chronic illnesses have reduced physiological reserve during acute illness.

Illness severity was strongly associated with mortality in both regression models. Higher SOFA and OASIS scores were associated with increased odds of death, supporting the value of these measures in risk stratification among critically ill patients. The SOFA score was developed to quantify the degree of organ dysfunction across multiple organ systems and has been widely used in intensive care settings [6]. Subsequent work has demonstrated the utility of organ dysfunction measures in identifying patients at increased risk of adverse outcomes [7]. The consistency of the findings across both severity measures strengthens confidence in the observed associations. The sensitivity analysis produced results similar to those obtained in the primary model, indicating that the association between documented hypertension and in-hospital mortality remained robust when illness severity was assessed using the OASIS score instead of the SOFA score.

The study also found that mechanical ventilation was associated with higher odds of in-hospital mortality. Mechanical ventilation is frequently required among critically ill patients with respiratory failure, severe infection, cardiovascular instability, or multisystem organ dysfunction. Patients requiring ventilatory support often represent a clinically complex subgroup with greater illness severity. Because mechanical ventilation may occur after ICU admission, it may also reflect progression of critical illness rather than solely a baseline patient characteristic. Accordingly, this association should be interpreted as a marker of illness severity rather than as a causal determinant of mortality. This observation is consistent with the marked differences in mortality according to ventilation status observed in the descriptive analysis. Age was associated with mortality in the primary model, consistent with previous studies reporting higher mortality rates among older ICU patients because of age-related reductions in physiological reserve and the accumulation of chronic disease [17].

Strengths and limitations of the study

A major strength of this study is the use of the MIMIC-IV database, which contains detailed clinical information from a large cohort of critically ill patients and enables comprehensive adjustment for important clinical factors [20,21]. The study incorporated validated measures of comorbidity burden and illness severity, including the CCI, SOFA score, and OASIS score.

Several limitations should also be considered. Information on blood pressure control before admission, duration of hypertension, antihypertensive medication use, treatment adherence, and outpatient disease management was not available. Data on lifestyle factors and socioeconomic characteristics were also unavailable. Documented hypertension was identified using ICD-9 and ICD-10 diagnosis codes recorded during the index hospitalization. Consequently, some patients may have had hypertension first documented during hospitalization rather than before ICU admission, and information on hypertension duration, severity, blood pressure control, and antihypertensive treatment was unavailable. In addition, the diagnostic codes included several hypertension subtypes, which may have introduced exposure heterogeneity and some degree of misclassification. The race or ethnicity category labeled “Unknown” may also reflect incomplete documentation or administrative factors rather than a true racial or ethnic group and should therefore be interpreted cautiously. Although missing data were minimal and unlikely to materially influence the findings, a complete-case approach was used. Finally, the retrospective observational design precludes causal inference, and residual confounding may remain despite multivariable adjustment. Future studies should examine the influence of hypertension severity, medication exposure, longitudinal blood pressure patterns, and cause-specific mortality outcomes among critically ill adults.

## Conclusions

This study highlights the association between documented hypertension and in-hospital mortality among critically ill adults admitted to ICUs. After accounting for demographic characteristics, comorbidity burden, illness severity, and mechanical ventilation, hypertension was associated with lower odds of in-hospital mortality. Age, comorbidity burden, illness severity, and mechanical ventilation were important factors associated with mortality risk. These findings demonstrate the importance of considering both chronic health conditions and acute clinical status when evaluating outcomes among critically ill patients. From a clinical perspective, comprehensive risk assessment remains essential for identifying patients at increased risk of adverse outcomes. Future research should examine the influence of hypertension severity, blood pressure control, antihypertensive treatment patterns, and longitudinal clinical trajectories to better understand outcomes among critically ill adults with hypertension.
